# Generation of bovine decellularized testicular bio-scaffolds as a 3D platform for testis bioengineering

**DOI:** 10.3389/fbioe.2024.1532107

**Published:** 2025-01-14

**Authors:** Francesca Di Filippo, Tiziana A. L. Brevini, Georgia Pennarossa, Fulvio Gandolfi

**Affiliations:** ^1^ Department of Agricultural and Environmental Sciences - Production, Landscape, Agroenergy, Università degli Studi di Milano, Milan, Italy; ^2^ Laboratory of Biomedical Embryology and Tissue Engineering, Department of Veterinary Medicine and Animal Sciences, Università degli Studi di Milano, Lodi, Italy

**Keywords:** 3D bio-scaffold, bovine, decellularization, extracellular matrix, testis

## Abstract

Accelerating the genetic selection to obtain animals more resilient to climate changes, and with a lower environmental impact, would greatly benefit by a substantial shortening of the generation interval. One way to achieve this goal is to generate male gametes directly from embryos. However, spermatogenesis is a complex biological process that, at present, can be partially reproduced *in vitro* only in the mouse. The development of reliable 3D *in vitro* models able to mimic the architecture and the physiological microenvironment of the testis, represents a possible strategy to facilitate *ex vivo* haploid male gamete generation in domestic species. Here we describe the creation of bovine testicular bio-scaffolds and their successful repopulation *in vitro* with bovine testicular cells. In particular, bovine testes are subjected to three different decellularization protocols. Cellular compartment removal and extracellular matrix preservation are evaluated. The generated bio-scaffolds are then repopulated with bovine testicular fibroblasts. The results obtained demonstrate that the decellularization protocol involving the use of 0.3% sodium dodecyl sulfate (SDS) for 12 h efficiently eliminates native cells, while preserving intact ECM composition and microstructure. Its subsequent repopulation with bovine fibroblasts demonstrates successful cell homing, colonization and growth, consistent with the scaffold ability to sustain cell adherence and proliferation. Overall, the generated 3D bio-scaffolds may constitute a suitable artificial niche for *ex vivo* culture of testicular cells and may represent a possible strategy to reproduce spermatogenesis *in vitro*.

## 1 Introduction

The obtainment of animals more resilient to climate changes, and with a lower environmental impact, would be greatly desirable. Based on this, during the last years, particular attention has been dedicated to the development and further improvement of a sustainable animal agriculture with positive socioeconomic and environmental impacts. In this perspective, new biotechnological approaches, such as assisted reproductive technologies (ART) and genetic selection (GS), were introduced and used simultaneously in livestock breeding programs, including in the bovine species (Mueller and Van Eenennaam, 2022). Nevertheless, the possibility of these techniques to improve genetic gain is limited by the average age of an animal when replacement offspring are born (Kasinathan et al., 2015). It is therefore clear that the greatest improvement in accelerating the genetic selection can be achieved by shortening the generation interval (Cenariu et al., 2012; Pasquariello et al., 2024). To this aim, different strategies have been proposed in both male and female domestic ruminants, however, a substantial shortening of the interval is still far away.

In the male, one promising approach is represented by the *in vitro* propagation and differentiation of spermatogonia into mature sperm or even *in vitro* recreation of whole spermatogenesis from embryonic stem cells. To date, a variety of culture systems as well as different medium compositions to enhance spermatogonial stem cell (SSC) viability, proliferation (Fath-Bayati et al., 2023; van Maaren et al., 2023) and differentiation *in vitro* (Cho and Easley, 2023; Kulibin and Malolina, 2023; Salem et al., 2023; Damyanova et al., 2024) have been developed. However, the complete spermatogenesis *ex vivo* has been obtained only in the murine species (Perrard et al., 2016), while, in cattle and in other domestic species, it is possible only to propagate spermatogonia without inducing an effective meiotic division. This is mainly due to the complexity of the process, during which male germ cells differentiate into mature spermatozoa, thank to well-orchestrated interactions among hormones, growth factors, cytokines, and extracellular matrix (ECM)-derived bio-mechanical and bio-chemical cues. In addition, the lack of knowledge on niche microenvironment, nutritional requirements, as well as on the multiple regulatory machinery driving self-renewal, proliferation, and differentiation, in ruminates, has significantly hindered progresses in this field. It is therefore desirable to developed reliable 3D *in vitro* models able to faithfully mimic the architecture and the physiological microenvironment of the native testicular tissue, bridging the gap between the *in vivo* complexity and the over-simplified conventional two-dimension (2D) *in vitro* cultures.

To date, several 3D platforms for testicular bioengineering, including testicular organoids (Richer et al., 2020), hydrogel bioreactors (Perrard et al., 2016), and synthetic, natural or decellularized scaffolds (Horvath-Pereira et al., 2023) have been developed in human, mouse and rat. In contrast, to our knowledge, no cell engrafted 3D scaffolds have been developed in the bovine species and only one study reported the differentiation of gonocytes into presumptive spermatids through the use the alginate encapsulation technique (Lee et al., 2001). In this scenario, the generation of a bovine decellularized testicular 3D scaffold represents a promising option, since it accurately replicates the *in vivo* topography and the complex milieu of the native tissue, thus promoting the necessary interactions between cells and their surrounding microenvironment. In addition, the preserved extracellular matrix (ECM), obtained through the decellularization process, provides essential biomechanical and biochemical cues that encourage the correct cell growth, differentiation, and function.

In the present study, we generate bovine testicular bio-scaffolds for the creation of reliable 3D artificial models. In particular, we test three different decellularization protocols and identify the protocol that better preserves intact ECM composition and microstructure, while efficiently eliminating cells. We then repopulate the generated testicular bio-scaffolds with fibroblasts isolated from bovine testes and monitor the scaffold ability to sustain cell adherence and proliferation.

## 2 Materials and methods

All reagents were purchased from Thermo Fisher Scientific unless otherwise indicated.

### 2.1 Ethic statement

Bovine testes were collected at the local abattoir from adult animals. Organs were isolated from animals destined to human consumption and, therefore, were not considered as animal experimentation under Directive 2010/63/EU of the European Parliament. All experiments were performed in accordance with the approved guidelines.

### 2.2 Testis collection

Organs were collected from four 2 years-old bulls at the local slaughterhouse and transported to the laboratory in cold sterile saline solution (NaCl 0.9%) within 1 hour. Testes were washed in phosphate-buffered saline (PBS), decapsulated and cut in small pieces of 0.5 × 0.5 × 0.5 cm³. Fragments were randomly allocated to four experimental groups: untreated tissue, control group, (CTR; n = 4), decellularization protocol A (Decell-A; n = 52); decellularization protocol B (Decell-B; n = 52), decellularization protocol C (Decell-C; n = 52). Untreated tissue samples, used ad control group (CTR), were immediately fixed in 10% buffered formalin (Bio-Optica) for histological evaluations. The other groups were subjected to the three different decellularization processes as described below.

### 2.3 Decellularization protocols

Testicular fragments belonging the experimental groups Decell-A, Decell-B, and Decell-C were frozen at −80°C for at least 24 h, thawed at 37°C in a water bath for 30 min, and decellularized in:

A. 0.3% (v/v) sodium dodecyl sulfate (SDS; Bio-Rad) in deionized water (DI-H2O) for 6 h and then in 1% (v/v) Triton X-100 in DI-H2O for 6 h;
B. 0.3% (v/v) sodium dodecyl sulfate (SDS; Bio-Rad) in DI-H2O for 12 h and then in 1% (v/v) Triton X-100 in DI-H2O for 6 h;
C. 0.3% (v/v) sodium dodecyl sulfate (SDS; Bio-Rad) DI-H2O for 24 h and then in 1% (v/v) Triton X-100 in DI-H2O for 6 h.

At the end of the decellularization protocols, testes were washed in DI-H2O for 6 h with changes every 2 h. All steps were carried out using an orbital shaker at 150 rpm at room temperature. At the end of the procedures, from each experimental group, samples were fixed for histology and stained with hematoxylin and eosin (H&E, Bio-Optica), Crossmon Trichrome (Bio-optica), Alcian blue (pH 1; Bio-optica) Orcein and 4,6-diamidino-2-phenylindole (DAPI), or used for *in vitro* re-seeding studies. Cell density analysis and stereological evaluations were then performed at least in triplicates.

### 2.4 Histological evaluations

Samples were fixed in 10% buffered formalin for 24 h at room temperature, dehydrated in graded alcohols, cleared with xylene, embedded in paraffin, and cut in serial microtome sections (5 μm thick). The latters were dewaxed, re-hydrated and stained with H&E (Bio-Optica), Crossmon Trichrome (Bio-optica), Alcian blue (pH 1; Bio-optica) and Orcein, in agreement with previously published studies (Verdile et al., 2022; Khazaei et al., 2023). Samples were analyzed under an Eclipse E600 microscope (Nikon) equipped with a digital camera (Nikon). Pictures were acquired with NIS-Elements Software (Version 4.6; Nikon). Untreated testicles were used as the control.

### 2.5 Cell density

Cell density analyses were carried out as previously described (Pennarossa et al., 2020). More in detail, serial microtome sections (5 μm thick) were cut, dewaxed, re-hydrated and stained with DAPI. Cell number was quantified in 5 tissue sections obtained from each testis (n = 4) subjected to the three different decellularization protocol (A, B, and C) and from 4 CTR testis. Within each section, 5 randomly selected fields at ×100 total magnifications were analyzed. Samples were analyzed under an Eclipse E600 microscope (Nikon) equipped with a digital camera (Nikon). Images were captured with NIS-Elements Software (Version 4.6; Nikon) and analyzed, using the Cell Counter plugin of the image analysis software ImageJ, following the instructions. Briefly, 8-bit grayscale images were generated applying threshold adjustments and segmented using a thresholding algorithm to highlight the areas occupied by the nuclei and remove the background. The data obtained were transformed into binary format. Size and circularity parameters were defined, and the nuclei were automatically enumerated. Untreated testicles were used as the control.

### 2.6 Stereological analyses

Collagen, elastin, and GAG volume density (Vv) evaluations were performed on sections stained with Crossmon Trichrome, Orcein and Alcian blue, respectively. As described by Albl et al. (2016), the Delesse principle was used, and the proportional volume of each specific area was calculated as the fraction of the structure of interest (e.g., collagen) relative to the total area of the reference compartment (e.g., whole section). Images were randomly taken, overlaid with a point-count stereological grid containing evenly spaced test points and the relative volume of each region of interest was calculated by dividing the number of points striking the structure of interest by the number of points hitting the reference compartment. Vv was expressed as percentages using the following formula:

$$\text{Vv }\left(\text{analyzed compartment},\text{reference compartment}\right)=\left[\sum P\left(\text{analyzed compartment}\right)/\sum P\left(\text{reference compartment}\right)\right]\times 100$$

∑ _P (analyzed compartment)_: the number of points hitting the compartment under study; ∑ _P (reference compartment)_: the number of points hitting the relevant structure.

### 2.7 Bovine testicular fibroblast isolation

Adult bovine fibroblasts were isolated from fresh testicular tissues obtained from 3 individuals (Albrecht et al., 2006). Testes were decapsulated and cut into small fragments of ∼ 2 mm³. These were placed into 35 mm^2^ Petri dishes (Sarstedt) previously coated with 0.1% gelatin (Sigma-Aldrich). Droplets of Dulbecco’s Modified Eagle Medium (DMEM) supplemented with 20% FBS, 2 mM glutamine (Sigma-Aldrich), and 2% antibiotic/antimycotic solution (Sigma-Aldrich) were added onto each fragment. Culture dishes were incubated in 5% CO2 at 37°C in humidified chambers. After 4 days of culture, bovine testicular fibroblasts started to grow out of the original explants, and the latter were carefully removed. Fibroblasts were cultured using the medium described above, grown in 5% CO2 at 37°C, and passaged twice a week at a 1:3 ratio. The three bovine primary cell lines were used in triplicate in 3 independent experiments.

### 2.8 Bio-scaffold repopulation with bovine testicular fibroblasts

Testicular bio-scaffolds obtained from Decell-A, Decell-B, and Decell-C groups were sterilized with 70% ethanol and 2% Penicillin/Streptomycin/Amphotericin B solution in sterile H_2_O for 30 min, extensively washed in sterile PBS and equilibrated in DMEM for 1 h at 37°C. Scaffolds of 0.5 × 0.5 cm^2^ and 1 mm thick were obtained using sharp scalpel and placed into a 4-well multidishes (1 fragment per well; Nunc). 0.5 × 10⁶ of bovine testicular fibroblasts were resuspended in 100 µL of DMEM supplemented with 10% fetal bovine serum (FBS), 2 mM glutamine (Sigma-Aldrich), and 1% antibiotic/antimycotic solution (Sigma-Aldrich), seeded onto each scaffold and co-cultured at 37°C incubator with 5% CO2. Re-seeding density was selected based on our previous studies (Pennarossa et al., 2020; Pennarossa et al., 2021b; Arcuri et al., 2024). Half medium volume was changed every 2 days. Cultures were arrested for histological evaluations at days 1, 3 and 7. All experiments were performed in triplicates.

### 2.9 Statistical analysis

Statistical analysis was performed using ANOVA with Tukey’s *post hoc* (SPSS 19.1; IBM). At least three experiments were carried out for all analyses. Data were reported as mean ± standard error of the mean (SEM). Differences of *p* ≤ 0.05 were considered significant.

## 3 Results

### 3.1 Testicular bio-scaffold evaluation

#### 3.1.1 Macroscopic assessments

**FIGURE 1:**
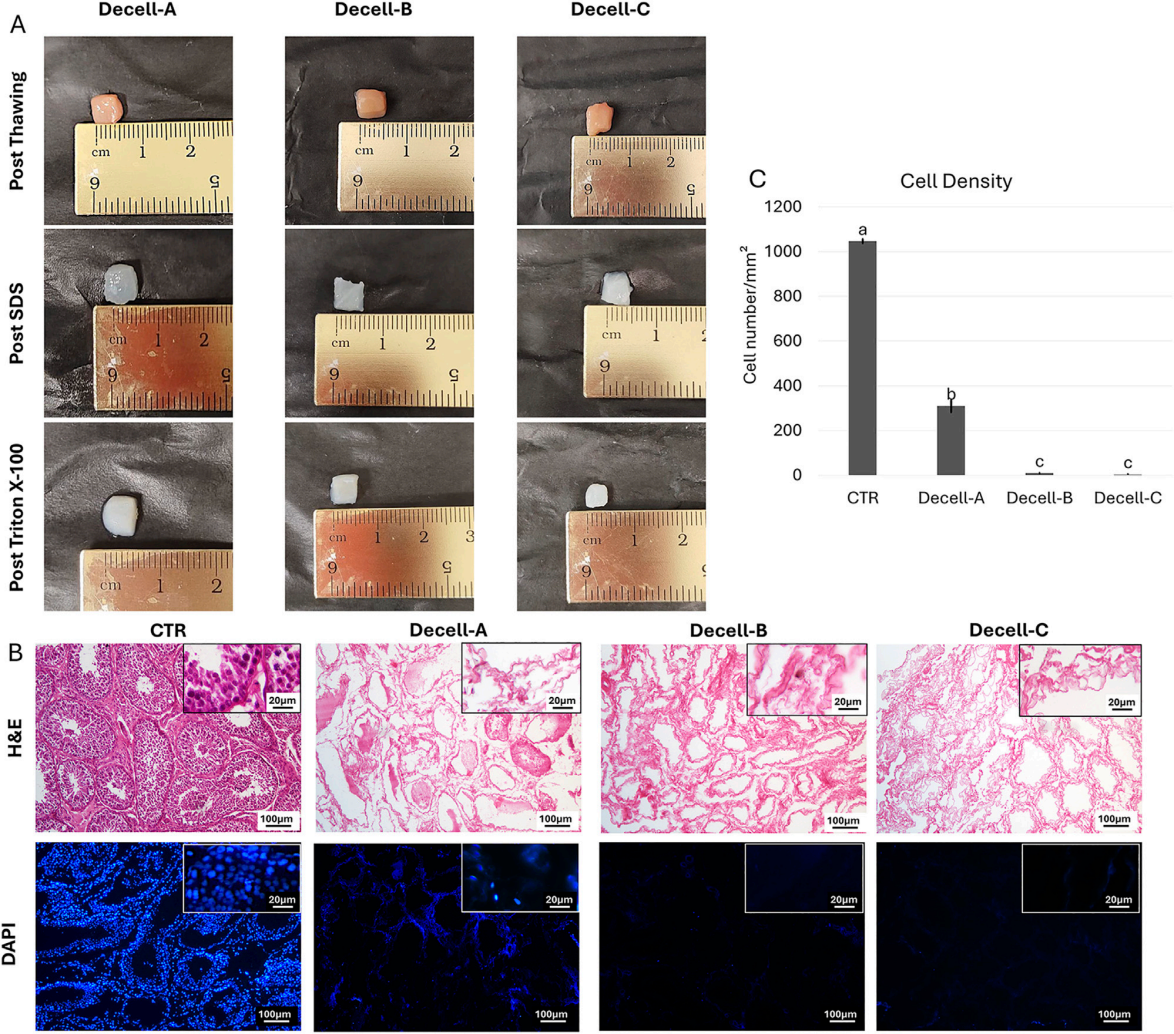
Macro/microscopic evaluations of decellularized testes. (A) Three-step macroscopic images illustrating the color of samples that turns from red to white, regardless of the decellularization protocol used. **(B)** H&E staining shows the presence of both basophilic (cell nuclei) and eosinophilic (cell cytoplasm and ECM) staining in untreated tissue (CTR) and their absence in decellularized testes. DAPI staining displaying the presence of nuclei in CTR samples and their disappearance after the decellularization processes. **(C)** Cell density analysis demonstrates a significantly lower number of nuclei in all decellularized tissues compared with the untreated one (CTR). Data are expressed as the mean ± standard error of the mean (SEM). A, b, c Different superscripts indicate *p* < 0.05.

Macroscopic observations revealed that, during the decellularization process, the color of the testicular fragments gradually turned from red to white, regardless to the decellularization protocol used (Decell-A, Decell-B, and Decell-C, Figure 1A).

#### 3.1.2 Histological analysis of cell nuclei and tissue after the different decellularization protocols

Histological assessments demonstrated that all the three decellularization protocols tested removed cells (Figure 1B). In particular, H&E staining showed the decrement of basophilic staining in Decell-A, Decell-B, and Decell-C (Figure 1B), while both the basophilic and eosinophilic staining were visible in the untreated tissue (CTR, Figure 1B). DAPI staining and cell density analysis confirmed these observations, indicating a significantly lower number of nuclei in all the obtained bio-scaffolds, when compared with the untreated tissues (Figures 1B, C). However, cell density analysis demonstrated that Decell-B and Decell-C protocol removed the cellular compartment more efficiently than Decell-A (Figure 1C).

**FIGURE 2:**
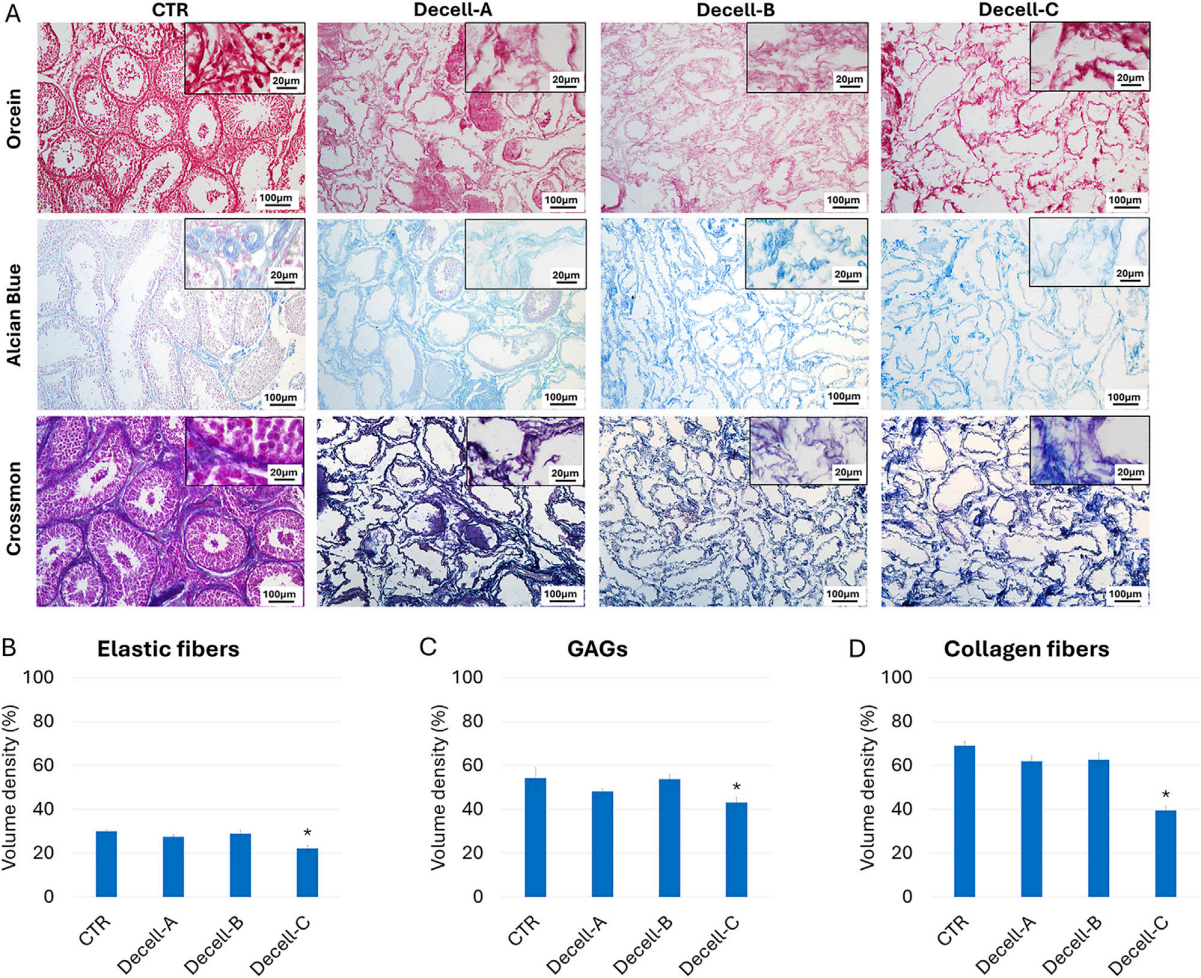
ECM microarchitecture and composition in untreated (CTR) and decellularized testes. **(A)** Orcein, alcian blue and Crossmon’s trichrome staining indicate the preservation of elastic fibers, GAGs, and collagen, respectively, in Decell-A and Decell-B, while a reduction for all the ECM components is visible in Decell-C. **(B)** Elastic fiber stereological quantification shows comparable amount of the protein in untreated tissue (CTR), Decell-A and Decell-B, while a significant decrement is detected in Decell-C. Data are expressed as the mean ± standard error of the mean (SEM) (*p > 0.05). **(C)** GAG stereological analysis displays a significant reduction in Decell-C compared to Decell-A, Decell-B and CTR. Data are expressed as the mean ± standard error of the mean (SEM) (*p > 0.05). **(D)** Collagen content quantification demonstrates comparable amount of the fibers in untreated tissue (CTR), Decell-A and Decell-B, while a significant decrement is detected in Decell-C. Data are expressed as the mean ± standard error of the mean (SEM) (*p > 0.05).

Orcein staining (Figure 2A) showed the preservation of intact elastic fibers after Decell-A and Decell-B, with a comparable distribution of elastin among Decell-A, Decell-B, and CTR samples. In contrast, a reduction was detected in Decell-C at the end of the process. These morphological observations were consistent with elastin stereological quantifications, which indicated a significant elastin decrement in the Decell-C group (Figure 2B). Similarly, Alcian Blue staining showed glycosaminoglycan (GAG) retention in Decell-A and Decell-B, which exhibited a GAG distribution comparable to that of the untreated tissue (CTR, Figure 2A). In contrast, a decrease was observed in Decell-C. Consistent with this, stereological analysis displayed statistically significant changes in total GAG content in Decell-C compared to the tissue of origin (Figure 2C). Crossmon trichrome staining demonstrated the persistence of collagen fibers in Decell-A and Decell-B samples, while a reduction was detected in Decell-C (Figure 2A). In agreement with these observations, stereological studies showed a statistically significant decrement of collagen fibers in Decell-C group when compared to CTR (Figure 2D).

### 3.2 Repopulation of the generated bio-scaffolds with bovine testicular fibroblasts

**FIGURE 3:**
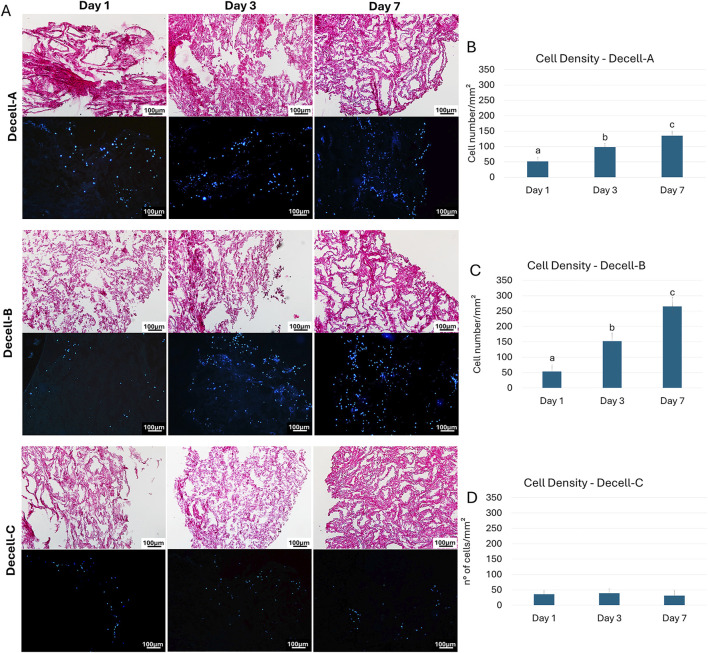
Re-seeding of decellularized testicular tissue. **(A)** H&E and DAPI staining demonstrate the presence of cells into the bio-scaffolds after 24 h from seeding (Day 1) in all the experimental groups. An increment during the following days (Day 3 and Day 7) is visible in Decell-A and Decell-B. **(B–D)** Cell density analyses confirm bio-scaffold re-population after 24 h (Day 1), with a significantly higher cell number at Day 3 and Day 7 in Decell-A and Decell-B. Decell-C shows comparable values at each time point considered. Data are expressed as the mean ± standard error of the mean (SEM). A, b, c, Different superscripts denote significant differences (*p* < 0.05).

Bovine testicular fibroblasts rapidly adhered and colonized Decell-A, Decell-B, and Decell-C within 24 h of co-culture (Figure 3A). H&E and DAPI staining showed an increasing number of cells during the following days for Decell-A and Decell-B, while no increment was visible in the Decell-C group (Figure 3A). These observations were further supported by cell density analysis that indicated the presence of cells into the bio-scaffolds 24 h after seeding (Day 1, Figures 3B–D) and a statistically significant cell number increment in Decell-A and Decell-B during the subsequent days of culture (Day 3 and Day 7, Figures 3B, C). No cell number increase was detected in the Decell-C during the 7-day culture period (Figure 3D).

## 4 Discussions

In the present manuscript, we generate a testicular bio-scaffold, able replicate the *in vivo* topography and the bio-mechanical and bio-chemical stimuli derived from the native ECM. The decellularized bio-scaffold successfully encourages cell homing, colonization and growth, demonstrating its ability to sustain testicular cell adherence and proliferation.

At the end of the three-step decellularization process, macroscopic evaluations revealed a color change from red to white in all the obtained bio-scaffolds, regardless of the protocol used. This suggests the occurrence of a significant reduction in the cellular components. Indeed, similar color variations were previously reported by other Authors, which applied decellularization protocols to different tissue, including heart (Rajabi-Zeleti et al., 2014), lung (Lecht et al., 2014), liver (Ghiringhelli et al., 2021; Lee et al., 2017), kidney (Yu et al., 2014), muscle (Aulino et al., 2015), trachea (Baiguera et al., 2014; Pennarossa et al., 2021b), esophagus (Sjöqvist et al., 2014), urinary tissue (Singh et al., 2018), arteries (Kajbafzadeh et al., 2017), derma (Gilpin and Yang, 2017), intestine (Arcuri et al., 2024), ovary (Laronda et al., 2015; Laronda, 2020; Pennarossa et al., 2020; Pennarossa et al., 2022) and vagina (Zhang et al., 2017), and that resulted in a significant decrease in cell content. This was confirmed by our H&E staining that demonstrated a decrease of basophilic color in all the three experimental groups and by DAPI staining, showing a significant decrement in cell nuclei in Decell-A, Decell-B, and Decell-C samples. All these morphological observations were further corroborated by cell density analysis experiments that indicated a statistically significant cell number reduction in all experimental groups, when compared with the untreated tissues (CTR). However, while the cellular compartment was reduced in all three groups, it is interesting to note that Decell-B and Decell-C protocols allowed a more efficient and significant reduction in cell number, compared to Decell-A. Altogether, these results demonstrate that the correct combination of a freeze-thaw cycle, with sequential incubations with SDS and Triton X-100, allows for an efficient cell removal only when the native tissue is exposed to SDS for at least 12 h, as in the protocols Decell-B and Decell-C. This is in agreement with previous studies which demonstrated SDS ability to successfully eliminate the cellular compartment from the native tissues (Scarrit et al., 2015; Singh et al., 2023), selecting in a tissue-specific manner the appropriate concentration and time of exposure (Gilpin and Yang, 2017).

It is however important to note that a fundamental aspect in the decellularization process is also the maintenance of the original ECM microstructures, including fibers and macromolecules. The histochemical analysis carried out in our study, demonstrated the preservation of intact elastic fibers, GAGs and collagen in Decell-A and Decell-B. This is in agreement with Kiani et al. (2021) and Khazaei et al. (2023) that described the generation of rat and calf testis scaffolds, respectively, and demonstrated the persistence of the major ECM proteins at the end of the decellularization process. In contrast, elastic and collagen fibers, as well as GAGs appeared to be significantly reduced when SDS exposure is prolonged (Decell-C). All these morphological observations were also confirmed by stereological analysis, which revealed no significant changes for collagen, elastin, and GAG content among the untreated tissue, Decell-A and Decell-B groups. In contrast, Decell-C showed a statistically significant decrement in the ECM components when compared to the CTR. Although several different explanations can be hypothesized, we suggest that the prolonged exposure to SDS used in the Decell-C protocol, while ensuring an efficient removal of the cellular compartment, may exert a detrimental effect and cause damages to structural proteins, such as collagen fibers and GAGs. This is in line with previous observations that described SDS disruptive side effects on collagen fibers in porcine urinary bladder (Faulk et al., 2014), caprine pancreas (Singh et al., 2023) and many other tissues (Crapo et al., 2011; Keane et al., 2015). It is also in agreement with Kasturi and Vasanthan and Moffat et al. that reported SDS ability to damage GAGs during the decellularization processes of different organs, including liver, pericardium, articular cartilage, heart, and kidney (Moffat et al., 2022; Kasturi and Vasanthan, 2023). All these observations clearly point to the need of a strategy that sets a fine tuning of the SDS conditions to ensure, in a species-specific and organ-specific way, the efficient removal of cells, while preserving an intact ECM structure.

A crucial point for the use of a bio-scaffold in tissue engineering is its ability to encourage cell adhesion, homing, and growth (Chan and Leong, 2008; Lynch et al., 2021; Pennarossa et al., 2021a). To address this point, we isolated fibroblasts from bovine testis and used them to repopulate the generated decellularized bio-scaffolds. The results obtained demonstrated a rapid engrafting process with cells that adhered and colonized the matrix within 24 h from seeding. In addition, H&E, DAPI staining and cell density analysis showed a linear increment in cell number and a homogenous and steady distribution of the cell population onto Decell-A and Decell-B scaffolds for as long as 7 days, when culture was arrested. It is also interesting to note that, although the number of cells identified in Decell-A should be affected by cellular residues present at the end of the decellularization process, the increasing number observed during the culture period demonstrates the presence of proliferating cells which derive from reseeding process. Altogether, these results indicate the bio-scaffold ability to host cells and to encourage their proliferation possibly via bio-mechanical and bio-chemical stimuli. They also exclude the persistence of toxic carry-overs from the decellularization processes, may impair the subsequent recellularization and biocompatibility, both *in vitro* and *in vivo* (Morris et al., 2017).

Overall, the results obtained suggest the potential of the bio-scaffold here described to sustain cell adherence and proliferation. In particular, the cell type selected for repopulation experiments well fits with the possible use of iPS-derived or chemically reprogrammed cells, for regenerative experiments of the testicular tissues. Although, these results are still preliminary, they pave the way toward the use of decellularized testicular bio-scaffolds in the field of reproductive biology and biotechnology as suitable artificial niches for *ex vivo* culture of testicular cells and to reproduce spermatogenesis *in vitro*.

## Data Availability

The raw data supporting the conclusions of this article will be made available by the authors, without undue reservation.

## References

[B1] Albl B. Haesner S. Braun-Reichhart C. Streckel E. Renner S. Seeliger F. (2016). Tissue sampling guides for porcine biomedical models. Toxicol. Pathol. 44, 414–420. 10.1177/0192623316631023 26883152

[B2] Albrecht M. Rämsch R. Köhn F. M. Schwarzer J. U. Mayerhofer A. (2006). Isolation and cultivation of human testicular peritubular cells: a new model for the investigation of fibrotic processes in the human testis and male infertility. J. Clin. Endocrinol. Metab. 91, 1956–1960. 10.1210/JC.2005-2169 16478819

[B3] Arcuri S. Pennarossa G. Prasadani M. Gandolfi F. Brevini T. A. L. (2024). Use of decellularized bio-scaffolds for the generation of a porcine artificial intestine. Methods Protoc. 7, 76. 10.3390/mps7050076 39452790 PMC11510128

[B4] Aulino P. Costa A. Chiaravalloti E. Perniconi B. Adamo S. Coletti D. (2015). Muscle extracellular matrix scaffold is a multipotent environment. Int. J. Med. Sci. 12, 336–340. 10.7150/ijms.10761 25897295 PMC4402437

[B5] Baiguera S. Del Gaudio C. Kuevda E. Gonfiotti A. Bianco A. Macchiarini P. (2014). Dynamic decellularization and cross-linking of rat tracheal matrix. Biomaterials 35, 6344–6350. 10.1016/J.BIOMATERIALS.2014.04.070 24818885

[B6] Cenariu M. Pall E. Cernea C. Groza I. (2012). Evaluation of bovine embryo biopsy techniques according to their ability to preserve embryo viability. J. Biomed. Biotechnol. 2012, 1–5. 10.1155/2012/541384 23091350 PMC3468301

[B7] Chan B. P. Leong K. W. (2008). Scaffolding in tissue engineering: general approaches and tissue-specific considerations. Eur. Spine J. 17, 467–479. 10.1007/s00586-008-0745-3 19005702 PMC2587658

[B8] Cho I. K. Easley C. A. (2023). Recent developments in *in vitro* spermatogenesis and future directions. Reprod. Med. 4, 215–232. 10.3390/REPRODMED4030020

[B9] Crapo P. M. Gilbert T. W. Badylak S. F. (2011). An overview of tissue and whole organ decellularization processes. Biomaterials 32, 3233–3243. 10.1016/j.biomaterials.2011.01.057 21296410 PMC3084613

[B10] Damyanova K. B. Nixon B. Johnston S. D. Gambini A. Benitez P. P. Lord T. (2024). Spermatogonial stem cell technologies: applications from human medicine to wildlife conservation. Biol. Reprod. 111, 757–779. 10.1093/BIOLRE/IOAE109 38993049 PMC11473898

[B11] Fath-Bayati L. Naserpour L. Khoshandam M. Jannatifar R. Fazaeli H. (2023). Recent advances in developing 3D culture systems of spermatogonial stem cell preservation and differentiation: a narrative review. Int. J. Reprod. Biomed. 21, 681–696. 10.18502/IJRM.V21I9.14397 37969562 PMC10643686

[B12] Faulk D. M. Carruthers C. A. Warner H. J. Kramer C. R. Reing J. E. Zhang L. (2014). The effect of detergents on the basement membrane complex of a biologic scaffold material. Acta Biomater. 10, 183–193. 10.1016/j.actbio.2013.09.006 24055455 PMC3857635

[B13] Ghiringhelli M. Abboud Y. Chorna S. V. Huber I. Arbel G. Gepstein A. (2021). Using decellularization/recellularization processes to prepare liver and cardiac engineered tissues. Methods Mol. Biol. 2273, 111–129. 10.1007/978-1-0716-1246-0_7 33604848

[B14] Gilpin A. Yang Y. (2017). Decellularization strategies for regenerative medicine: from processing techniques to applications. Biomed. Res. Int. 2017, 1–13. 10.1155/2017/9831534 PMC5429943 28540307

[B15] Horvath-Pereira B. de O. Almeida G. H. D. R. Silva Júnior L. N. da do Nascimento P. G. Horvath Pereira B. de O. Fireman J. V. B. T. (2023). Biomaterials for Testicular Bioengineering: how far have we come and where do we have to go? Front. Endocrinol. (Lausanne) 14, 1085872. 10.3389/fendo.2023.1085872 37008920 PMC10060902

[B16] Kajbafzadeh A.-M. Khorramirouz R. Kameli S. M. Hashemi J. Bagheri A. (2017). Decellularization of human internal mammary artery: biomechanical properties and histopathological evaluation. Biores Open Access 6, 74–84. 10.1089/biores.2016.0040 28736690 PMC5515095

[B17] Kasinathan P. Wei H. Xiang T. Molina J. A. Metzger J. Broek D. (2015). Acceleration of genetic gain in cattle by reduction of generation interval. Sci. Rep. 5, 8674–4. 10.1038/srep08674 25728468 PMC4345332

[B18] Kasturi M. Vasanthan K. S. (2023). Effect of decellularization using sodium dodecyl sulfate on glycosaminoglycans content in the liver. Regen. Med. 18, 527–530. 10.2217/rme-2023-0050 37350181

[B19] Keane T. J. Swinehart I. T. Badylak S. F. (2015). Methods of tissue decellularization used for preparation of biologic scaffolds and *in vivo* relevance. Methods 84, 25–34. 10.1016/j.ymeth.2015.03.005 25791470

[B20] Khazaei M. R. Ami Z. Khazaei M. Rezakhani L. (2023). The decellularized calf testis: introducing suitable scaffolds for spermatogenesis studies. Int. J. Fertil. Steril. 18, 32–39. 10.22074/IJFS.2023.1989173.1433 38041457 PMC10692750

[B21] Kiani M. Movahedin M. Halvaei I. Soleimani M. (2021). Formation of organoid-like structures in the decellularized rat testis. Iran. J. Basic Med. Sci. 24, 1523–1528. 10.22038/IJBMS.2021.58294.12948 35317108 PMC8917852

[B22] Kulibin A. Y. Malolina E. A. (2023). *In vitro* spermatogenesis: in search of fully defined conditions. Front. Cell Dev. Biol. 11, 1106111. 10.3389/FCELL.2023.1106111 36910153 PMC9998899

[B23] Laronda M. M. (2020). Engineering a bioprosthetic ovary for fertility and hormone restoration. Theriogenology 150, 8–14. 10.1016/j.theriogenology.2020.01.021 31973967

[B24] Laronda M. M. Jakus A. E. Whelan K. A. Wertheim J. A. Shah R. N. Woodruff T. K. (2015). Initiation of puberty in mice following decellularized ovary transplant. Biomaterials 50, 20–29. 10.1016/j.biomaterials.2015.01.051 25736492 PMC4350019

[B25] Lecht S. Stabler C. T. Rylander A. L. Chiaverelli R. Schulman E. S. Marcinkiewicz C. (2014). Enhanced reseeding of decellularized rodent lungs with mouse embryonic stem cells. Biomaterials 35, 3252–3262. 10.1016/j.biomaterials.2013.12.093 24439414 PMC5030820

[B26] Lee D. R. Kaproth M. T. Parks J. E. (2001). *In vitro* production of haploid germ cells from fresh or frozen-thawed testicular cells of neonatal bulls 1. Biol. Reprod. 65, 873–878. 10.1095/biolreprod65.3.873 11514353

[B27] Lee H. Han W. Kim H. Ha D.-H. Jang J. Kim B. S. (2017). Development of liver decellularized extracellular matrix bioink for three-dimensional cell printing-based liver tissue engineering. Biomacromolecules 18, 1229–1237. 10.1021/acs.biomac.6b01908 28277649

[B28] Lynch C. R. Kondiah P. P. D. Choonara Y. E. (2021). Advanced strategies for tissue engineering in regenerative medicine: a biofabrication and biopolymer perspective. Molecules 26, 2518. 10.3390/molecules26092518 33925886 PMC8123515

[B29] Moffat D. Ye K. Jin S. (2022). Decellularization for the retention of tissue niches. J. Tissue Eng. 13, 20417314221101151. 10.1177/20417314221101151 35620656 PMC9128068

[B30] Morris A. H. Stamer D. K. Kyriakides T. R. (2017). The host response to naturally-derived extracellular matrix biomaterials. Semin. Immunol. 29, 72–91. 10.1016/J.SMIM.2017.01.002 28274693

[B31] Mueller M. L. Van Eenennaam A. L. (2022). Synergistic power of genomic selection, assisted reproductive technologies, and gene editing to drive genetic improvement of cattle. CABI Agric. Biosci. 3, 13–29. 10.1186/S43170-022-00080-Z

[B32] Pasquariello R. Bogliolo L. Di Filippo F. Leoni G. G. Nieddu S. Podda A. (2024). Use of assisted reproductive technologies (ARTs) to shorten the generational interval in ruminants: current status and perspectives. Theriogenology 225, 16–32. 10.1016/J.THERIOGENOLOGY.2024.05.026 38788626

[B33] Pennarossa G. De Iorio T. Gandolfi F. Brevini T. A. L. (2021a). Ovarian decellularized bioscaffolds provide an optimal microenvironment for cell growth and differentiation *in vitro* . Cells 10, 2126. 10.3390/CELLS10082126 34440895 PMC8393799

[B34] Pennarossa G. De Iorio T. Gandolfi F. Brevini T. A. L. (2022). Impact of aging on the ovarian extracellular matrix and derived 3D scaffolds. Nanomater. (Basel) 12, 345. 10.3390/NANO12030345 PMC8839021 35159690

[B35] Pennarossa G. Ghiringhelli M. Gandolfi F. Brevini T. A. L. (2020). Whole-ovary decellularization generates an effective 3D bioscaffold for ovarian bioengineering. J. Assist. Reprod. Genet. 37, 1329–1339. 10.1007/s10815-020-01784-9 32361917 PMC7311562

[B36] Pennarossa G. Ghiringhelli M. Gandolfi F. Brevini T. A. L. (2021b). Tracheal *in vitro* reconstruction using a decellularized bio-scaffold in combination with a rotating bioreactor. Methods Mol. Biol. 2436, 157–165. 10.1007/7651_2021_398 33950378

[B37] Perrard M. H. Sereni N. Schluth-Bolard C. Blondet A. Giscard d’Estaing S. Plotton I. (2016). Complete human and rat *ex vivo* spermatogenesis from fresh or frozen testicular tissue. Biol. Reprod. 95, 89–90. 10.1095/biolreprod.116.142802 27580986

[B38] Rajabi-Zeleti S. Jalili-Firoozinezhad S. Azarnia M. Khayyatan F. Vahdat S. Nikeghbalian S. (2014). The behavior of cardiac progenitor cells on macroporous pericardium-derived scaffolds. Biomaterials 35, 970–982. 10.1016/J.BIOMATERIALS.2013.10.045 24183165

[B39] Richer G. Baert Y. Goossens E. (2020). *In-vitro* spermatogenesis through testis modelling: toward the generation of testicular organoids. Andrology 8, 879–891. 10.1111/ANDR.12741 31823507 PMC7496450

[B40] Salem M. Khadivi F. Javanbakht P. Mojaverrostami S. Abbasi M. Feizollahi N. (2023). Advances of three-dimensional (3D) culture systems for *in vitro* spermatogenesis. Stem Cell Res. Ther. 14, 262–330. 10.1186/s13287-023-03466-6 37735437 PMC10512562

[B41] Scarrit M. E. Pashos N. C. Bunnell B. A. (2015). A review of cellularization strategies for tissue engineering of whole organs. Front. Bioeng. Biotechnol. 3, 43. 10.3389/fbioe.2015.00043 25870857 PMC4378188

[B42] Singh A. Bivalacqua T. J. Sopko N. (2018). Urinary tissue engineering: challenges and opportunities. Sex. Med. Rev. 6, 35–44. 10.1016/J.SXMR.2017.08.004 29066225

[B43] Singh G. Satpathi S. Gopala Reddy B. V. Singh M. K. Sarangi S. Behera P. K. (2023). Impact of various detergent-based immersion and perfusion decellularization strategies on the novel caprine pancreas derived extracellular matrix scaffold. Front. Bioeng. Biotechnol. 11, 1253804. 10.3389/fbioe.2023.1253804 37790257 PMC10544968

[B44] Sjöqvist S. Jungebluth P. Lim M. L. Haag J. C. Gustafsson Y. Lemon G. (2014). Experimental orthotopic transplantation of a tissue-engineered oesophagus in rats. Nat. Commun. 5, 3562. 10.1038/ncomms4562 24736316 PMC4354271

[B45] van Maaren J. Alves L. F. van Wely M. van Pelt A. M. M. Mulder C. L. (2023). Favorable culture conditions for spermatogonial propagation in human and non-human primate primary testicular cell cultures: a systematic review and meta-analysis. Front. Cell Dev. Biol. 11, 1330830. 10.3389/fcell.2023.1330830 38259514 PMC10800969

[B46] Verdile N. Pasquariello R. Cardinaletti G. Tibaldi E. Brevini T. A. L. Gandolfi F. (2022). Telocytes: active players in the rainbow trout (oncorhynchus mykiss) intestinal stem-cell niche. Animals 12, 74. 10.3390/ani12010074 PMC8744786 35011180

[B47] Yu Y. L. Shao Y. K. Ding Y. Q. Lin K. Z. Chen B. Zhang H. Z. (2014). Decellularized kidney scaffold-mediated renal regeneration. Biomaterials 35, 6822–6828. 10.1016/j.biomaterials.2014.04.074 24855960

[B48] Zhang J.-K. Du R.-X. Zhang L. Li Y.-N. Zhang M.-L. Zhao S. (2017). A new material for tissue engineered vagina reconstruction: acellular porcine vagina matrix. J. Biomed. Mater Res. A 105, 1949–1959. 10.1002/jbm.a.36066 28294563

